# Supplementary material for: Pyruvate Dehydrogenase Kinase 1 inhibition mediated oxidative phosphorylation enhancement in cartilage promotes osteoarthritis progression
Source: BMC Musculoskelet Disord. 2023 Jul 20;24:597. doi: 10.1186/s12891-023-06585-6 (PMC10357736; doi:10.1186/s12891-023-06585-6)
Supplement: Supplementary file 1 — Additional file 1. [file 12891_2023_6585_MOESM1_ESM.zip › KEGG.Gsea.1653622432464/KEGG_ADIPOCYTOKINE_SIGNALING_PATHWAY.html]

Details for gene set KEGG\_ADIPOCYTOKINE\_SIGNALING\_PATHWAY[GSEA]

|  || Dataset | 55457\_collapsed\_to\_symbols.cls.cls #OA\_versus\_normal.cls.cls #OA\_versus\_normal\_repos |
| Phenotype | cls.cls#OA\_versus\_normal\_repos |
| Upregulated in class | normal |
| GeneSet | KEGG\_ADIPOCYTOKINE\_SIGNALING\_PATHWAY |
| Enrichment Score (ES) | -0.3904127 |
| Normalized Enrichment Score (NES) | -1.5666755 |
| Nominal p-value | 0.007886435 |
| FDR q-value | 0.16754569 |
| FWER p-Value | 0.755 |
Table: GSEA Results Summary

  

Fig 1: Enrichment plot: KEGG\_ADIPOCYTOKINE\_SIGNALING\_PATHWAY      
 Profile of the Running ES Score & Positions of GeneSet Members on the Rank Ordered List

  

| SYMBOL | TITLE | RANK IN GENE LIST | RANK METRIC SCORE | RUNNING ES | CORE ENRICHMENT || 1 | PRKAG1 | protein kinase AMP-activated non-catalytic subunit gamma 1 [Source:HGNC Symbol;Acc:HGNC:9385] | 138 | 0.870 | 0.0280 | No |
| 2 | ADIPOR1 | adiponectin receptor 1 [Source:HGNC Symbol;Acc:HGNC:24040] | 368 | 0.718 | 0.0422 | No |
| 3 | NFKBIE | NFKB inhibitor epsilon [Source:HGNC Symbol;Acc:HGNC:7799] | 626 | 0.613 | 0.0495 | No |
| 4 | MAPK9 | mitogen-activated protein kinase 9 [Source:HGNC Symbol;Acc:HGNC:6886] | 721 | 0.588 | 0.0683 | No |
| 5 | PCK2 | "phosphoenolpyruvate carboxykinase 2, mitochondrial [Source:HGNC Symbol;Acc:HGNC:8725]" | 759 | 0.579 | 0.0912 | No |
| 6 | AKT1 | AKT serine/threonine kinase 1 [Source:HGNC Symbol;Acc:HGNC:391] | 1192 | 0.478 | 0.0789 | No |
| 7 | TNF | tumor necrosis factor [Source:HGNC Symbol;Acc:HGNC:11892] | 2098 | 0.350 | 0.0240 | No |
| 8 | CAMKK2 | calcium/calmodulin dependent protein kinase kinase 2 [Source:HGNC Symbol;Acc:HGNC:1470] | 2497 | 0.303 | 0.0065 | No |
| 9 | PRKCQ | protein kinase C theta [Source:HGNC Symbol;Acc:HGNC:9410] | 3658 | 0.189 | -0.0755 | No |
| 10 | RELA | "RELA proto-oncogene, NF-kB subunit [Source:HGNC Symbol;Acc:HGNC:9955]" | 3942 | 0.161 | -0.0903 | No |
| 11 | JAK2 | Janus kinase 2 [Source:HGNC Symbol;Acc:HGNC:6192] | 4183 | 0.140 | -0.1028 | No |
| 12 | PRKAG2 | protein kinase AMP-activated non-catalytic subunit gamma 2 [Source:HGNC Symbol;Acc:HGNC:9386] | 4362 | 0.123 | -0.1112 | No |
| 13 | NFKB1 | nuclear factor kappa B subunit 1 [Source:HGNC Symbol;Acc:HGNC:7794] | 4626 | 0.102 | -0.1271 | No |
| 14 | G6PC1 | glucose-6-phosphatase catalytic subunit 1 [Source:HGNC Symbol;Acc:HGNC:4056] | 5389 | 0.041 | -0.1847 | No |
| 15 | TRADD | TNFRSF1A associated via death domain [Source:HGNC Symbol;Acc:HGNC:12030] | 5713 | 0.015 | -0.2092 | No |
| 16 | IKBKB | inhibitor of nuclear factor kappa B kinase subunit beta [Source:HGNC Symbol;Acc:HGNC:5960] | 5914 | 0.001 | -0.2247 | No |
| 17 | IRS4 | insulin receptor substrate 4 [Source:HGNC Symbol;Acc:HGNC:6128] | 6106 | -0.013 | -0.2390 | No |
| 18 | ACSL6 | acyl-CoA synthetase long chain family member 6 [Source:HGNC Symbol;Acc:HGNC:16496] | 6505 | -0.043 | -0.2681 | No |
| 19 | ADIPOR2 | adiponectin receptor 2 [Source:HGNC Symbol;Acc:HGNC:24041] | 6729 | -0.058 | -0.2829 | No |
| 20 | RXRG | retinoid X receptor gamma [Source:HGNC Symbol;Acc:HGNC:10479] | 6947 | -0.075 | -0.2964 | No |
| 21 | SLC2A1 | solute carrier family 2 member 1 [Source:HGNC Symbol;Acc:HGNC:11005] | 7089 | -0.087 | -0.3036 | No |
| 22 | RXRA | retinoid X receptor alpha [Source:HGNC Symbol;Acc:HGNC:10477] | 7381 | -0.109 | -0.3214 | No |
| 23 | ADIPOQ | "adiponectin, C1Q and collagen domain containing [Source:HGNC Symbol;Acc:HGNC:13633]" | 7792 | -0.141 | -0.3470 | No |
| 24 | NPY | neuropeptide Y [Source:HGNC Symbol;Acc:HGNC:7955] | 7834 | -0.143 | -0.3439 | No |
| 25 | CD36 | CD36 molecule [Source:HGNC Symbol;Acc:HGNC:1663] | 7985 | -0.154 | -0.3487 | No |
| 26 | AKT3 | AKT serine/threonine kinase 3 [Source:HGNC Symbol;Acc:HGNC:393] | 8008 | -0.156 | -0.3435 | No |
| 27 | CPT1A | carnitine palmitoyltransferase 1A [Source:HGNC Symbol;Acc:HGNC:2328] | 8046 | -0.159 | -0.3393 | No |
| 28 | ACSL3 | acyl-CoA synthetase long chain family member 3 [Source:HGNC Symbol;Acc:HGNC:3570] | 8170 | -0.169 | -0.3413 | No |
| 29 | TNFRSF1A | TNF receptor superfamily member 1A [Source:HGNC Symbol;Acc:HGNC:11916] | 8196 | -0.171 | -0.3357 | No |
| 30 | TRAF2 | TNF receptor associated factor 2 [Source:HGNC Symbol;Acc:HGNC:12032] | 8322 | -0.180 | -0.3374 | No |
| 31 | STAT3 | signal transducer and activator of transcription 3 [Source:HGNC Symbol;Acc:HGNC:11364] | 8453 | -0.190 | -0.3391 | No |
| 32 | LEPR | leptin receptor [Source:HGNC Symbol;Acc:HGNC:6554] | 8479 | -0.192 | -0.3325 | No |
| 33 | PRKAB1 | protein kinase AMP-activated non-catalytic subunit beta 1 [Source:HGNC Symbol;Acc:HGNC:9378] | 8708 | -0.210 | -0.3409 | No |
| 34 | LEP | leptin [Source:HGNC Symbol;Acc:HGNC:6553] | 8740 | -0.212 | -0.3339 | No |
| 35 | PRKAA2 | protein kinase AMP-activated catalytic subunit alpha 2 [Source:HGNC Symbol;Acc:HGNC:9377] | 8864 | -0.222 | -0.3335 | No |
| 36 | MAPK8 | mitogen-activated protein kinase 8 [Source:HGNC Symbol;Acc:HGNC:6881] | 8956 | -0.228 | -0.3305 | No |
| 37 | G6PC2 | glucose-6-phosphatase catalytic subunit 2 [Source:HGNC Symbol;Acc:HGNC:28906] | 9088 | -0.239 | -0.3300 | No |
| 38 | PCK1 | phosphoenolpyruvate carboxykinase 1 [Source:HGNC Symbol;Acc:HGNC:8724] | 9100 | -0.239 | -0.3202 | No |
| 39 | ACSL5 | acyl-CoA synthetase long chain family member 5 [Source:HGNC Symbol;Acc:HGNC:16526] | 9136 | -0.243 | -0.3121 | No |
| 40 | MAPK10 | mitogen-activated protein kinase 10 [Source:HGNC Symbol;Acc:HGNC:6872] | 9699 | -0.291 | -0.3429 | No |
| 41 | CPT1B | carnitine palmitoyltransferase 1B [Source:HGNC Symbol;Acc:HGNC:2329] | 10179 | -0.339 | -0.3652 | No |
| 42 | PPARGC1A | PPARG coactivator 1 alpha [Source:HGNC Symbol;Acc:HGNC:9237] | 10504 | -0.374 | -0.3738 | Yes |
| 43 | ACSL1 | acyl-CoA synthetase long chain family member 1 [Source:HGNC Symbol;Acc:HGNC:3569] | 10705 | -0.394 | -0.3718 | Yes |
| 44 | SLC2A4 | solute carrier family 2 member 4 [Source:HGNC Symbol;Acc:HGNC:11009] | 10764 | -0.400 | -0.3585 | Yes |
| 45 | ACACB | acetyl-CoA carboxylase beta [Source:HGNC Symbol;Acc:HGNC:85] | 10955 | -0.425 | -0.3543 | Yes |
| 46 | TNFRSF1B | TNF receptor superfamily member 1B [Source:HGNC Symbol;Acc:HGNC:11917] | 10969 | -0.427 | -0.3363 | Yes |
| 47 | IKBKG | inhibitor of nuclear factor kappa B kinase regulatory subunit gamma [Source:HGNC Symbol;Acc:HGNC:5961] | 10986 | -0.428 | -0.3185 | Yes |
| 48 | AGRP | agouti related neuropeptide [Source:HGNC Symbol;Acc:HGNC:330] | 10994 | -0.429 | -0.3000 | Yes |
| 49 | POMC | proopiomelanocortin [Source:HGNC Symbol;Acc:HGNC:9201] | 11010 | -0.431 | -0.2819 | Yes |
| 50 | PPARA | peroxisome proliferator activated receptor alpha [Source:HGNC Symbol;Acc:HGNC:9232] | 11110 | -0.445 | -0.2698 | Yes |
| 51 | PTPN11 | protein tyrosine phosphatase non-receptor type 11 [Source:HGNC Symbol;Acc:HGNC:9644] | 11260 | -0.465 | -0.2607 | Yes |
| 52 | NFKBIB | NFKB inhibitor beta [Source:HGNC Symbol;Acc:HGNC:7798] | 11277 | -0.468 | -0.2411 | Yes |
| 53 | ACSL4 | acyl-CoA synthetase long chain family member 4 [Source:HGNC Symbol;Acc:HGNC:3571] | 11327 | -0.475 | -0.2238 | Yes |
| 54 | IRS2 | insulin receptor substrate 2 [Source:HGNC Symbol;Acc:HGNC:6126] | 11390 | -0.485 | -0.2070 | Yes |
| 55 | STK11 | serine/threonine kinase 11 [Source:HGNC Symbol;Acc:HGNC:11389] | 11451 | -0.496 | -0.1896 | Yes |
| 56 | PRKAA1 | protein kinase AMP-activated catalytic subunit alpha 1 [Source:HGNC Symbol;Acc:HGNC:9376] | 11499 | -0.501 | -0.1709 | Yes |
| 57 | CHUK | component of inhibitor of nuclear factor kappa B kinase complex [Source:HGNC Symbol;Acc:HGNC:1974] | 11624 | -0.521 | -0.1574 | Yes |
| 58 | IRS1 | insulin receptor substrate 1 [Source:HGNC Symbol;Acc:HGNC:6125] | 11841 | -0.557 | -0.1494 | Yes |
| 59 | RXRB | retinoid X receptor beta [Source:HGNC Symbol;Acc:HGNC:10478] | 11871 | -0.565 | -0.1265 | Yes |
| 60 | MTOR | mechanistic target of rapamycin kinase [Source:HGNC Symbol;Acc:HGNC:3942] | 12047 | -0.605 | -0.1131 | Yes |
| 61 | SOCS3 | suppressor of cytokine signaling 3 [Source:HGNC Symbol;Acc:HGNC:19391] | 12453 | -0.725 | -0.1124 | Yes |
| 62 | PRKAB2 | protein kinase AMP-activated non-catalytic subunit beta 2 [Source:HGNC Symbol;Acc:HGNC:9379] | 12731 | -0.893 | -0.0942 | Yes |
| 63 | NFKBIA | NFKB inhibitor alpha [Source:HGNC Symbol;Acc:HGNC:7797] | 12817 | -1.001 | -0.0562 | Yes |
| 64 | AKT2 | AKT serine/threonine kinase 2 [Source:HGNC Symbol;Acc:HGNC:392] | 12896 | -1.404 | 0.0003 | Yes |
Table: GSEA details [plain text format]

  

Fig 2: KEGG\_ADIPOCYTOKINE\_SIGNALING\_PATHWAY      
 Blue-Pink O' Gram in the Space of the Analyzed GeneSet

  

Fig 3: KEGG\_ADIPOCYTOKINE\_SIGNALING\_PATHWAY: Random ES distribution      
 Gene set null distribution of ES for **KEGG\_ADIPOCYTOKINE\_SIGNALING\_PATHWAY**
